# Protective factors for suicidal ideation and suicide attempts in adolescence: a longitudinal population-based cohort study examining sex differences

**DOI:** 10.1186/s12888-025-06552-6

**Published:** 2025-02-06

**Authors:** Victoria Bakken, Stian Lydersen, Norbert Skokauskas, Anne Mari Sund, Jannike Kaasbøll

**Affiliations:** Department of Mental Health, Norwegian University of Science and Technology (NTNU), Trondheim, 7491 Norway

**Keywords:** Adolescence, Protective factors, Suicidal ideation, Suicide attempts, Self-worth

## Abstract

**Background:**

Adolescence is a critical period with elevated vulnerability to suicidality. Despite the importance of protective factors in reducing suicidal ideation (SI) and suicide attempts (SA), research in this area remains limited. This study investigated the associations between protective factors in early adolescence and the subsequent outcomes of SI and SA a year later, while also examining sex differences in these associations.

**Methods:**

This study utilized data from a representative sample at two timepoints, T1 (*N* = 2464, 50.8% females, mean age = 13.7, SD = 0.6) in 1998 and T2 (*N* = 2432, 50.4% females, mean age = 14.9, SD = 0.6) one year later, collected as part of the longitudinal Youth and Mental Health Study (YAMHS) in Norway. Individual, social and environmental protective factors were identified based on prior research and theoretical frameworks. We used linear (or ordinal logistic) regression analyses with SI (or SA) as dependent variable, and one protective factor, sex and their interaction as covariates.

**Results:**

Positive self-perceptions (T1) were significantly associated with reduced SI and SA one year later (T2) for both sexes. Specifically, self-worth was more strongly associated with reduced SI in females (*B* = -0.16, 95% CI: -0.20 to -0.12, *p* <.001) than males (*B* = -0.08, 95% CI: -0.12 to -0.04, *p* <.001). The interaction between sex and social competence influenced the likelihood of SA, with males (OR = 0.24, 95% CI: 0.13 to 0.42, *p* <.001) showing a greater reduction than females (OR = 0.53, 95% CI: 0.35 to 0.80, *p* =.009), although this association only showed a trend towards significance (*p* =.083). Higher physical activity and school connectedness/wellbeing were associated with lower SI, and school connectedness/wellbeing to lower SA, with no sex differences. No significant associations were found between task-oriented coping, attachment, family functioning or socio-economic status and SI or SA.

**Conclusions:**

Fostering positive self-perception, physical activity, and school connectedness/wellbeing is important for adolescent suicide prevention, as these factors contribute to reducing suicidality. Sex differences were observed in the associations between self-worth and SI, as well as social competence and SA. Future research should explore additional protective factors across sex and gender groups.

**Supplementary Information:**

The online version contains supplementary material available at 10.1186/s12888-025-06552-6.

## Introduction

### Suicidality in adolescence

Suicide is a global health concern and the fourth leading cause of death among adolescents [1]. Suicidality is defined as an increased risk of suicide, that may involve ideations, intent, plans, or suicidal behaviors such as suicide attempts [2]. Suicidal ideations (SI) are thoughts or contemplations about suicide [3], and suicide attempts (SA) is usually defined as surviving trying to end one’s own life [4]. Investigating SI and SA both independently and in conjunction is essential, as they represent distinct yet interconnected stages on the suicidality spectrum, each with unique risk factors, implications, and intervention strategies [5]. A recent meta-analysis identified an international prevalence of 14–22% for SI and 4–15% for SA among community adolescents [6]. Most adolescents do not act on SI [7], yet SI is a robust precursor for SA [8] and history of SA is a prominent risk factor for future attempts or completed suicide [9, 10].

Adolescence is a high-risk period characterized by a range of physical, psychological, and social developmental changes that increase potential vulnerabilities to common mental health challenges, including suicidality [11–14] Several gender/sex-specific risks have been identified in suicidality, also referred to as the “gender paradox” whereby females report more SI and SA, yet males have the highest rates of completed suicide [15]. A longitudinal study found that externalizing problems, depression and social contexts influenced trajectories in SI among both sexes in early adolescence [16]. Although males with initial high SI steadily declined over time, SI increased between age 12–13 in females before decreasing after age 14–15. These findings point to early adolescence as a critical period for addressing suicidality, given the variability in SI patterns. A meta-analysis of longitudinal studies identified that common risk factors for SA included mental health disorders, history of abuse and interpersonal problems in females, while behavior problems, parents’ relationship-dissolution, access to means and exposure to friends’ suicide were risk factors for males [17]. Adolescent suicidality is also linked to long-term mental health challenges and increased suicide risk in adulthood [18], with suicide mortality remaining highest among males [19]. Despite efforts to identify and mitigate risk factors, suicide rates have remained relatively stable for decades, suggesting that alternative approaches are necessary [20, 21]. Compared to risk factors for suicidality, the research on protective factors remains sparse [17, 22, 23]. The World Health Organization (WHO) asserted that while reducing risk-factors have been the primary focus of prevention efforts, enhancing knowledge of protective factors is equally important [14]. Hence, identifying and understanding protective factors for SI and SA during adolescence is crucial for early and effective suicide prevention.

### Protective factors for suicidality in adolescence

Protective factors are characteristics, conditions, or behaviors that decrease the likelihood of suicidality, encompassing individual, relational, societal, and contextual elements [24, 25]. In the context of adolescent development, incorporating the ecological perspective is essential [23, 26]. The conceptual framework of this study is based on the protective factor model, whereby promotive factors are referred to as protective factors to detach them from specific counterpart risk factors, involving processes that reduce risk-effects in negative outcomes [27].

A recent literature review analyzing 26 studies on protective factors for SA in children, adolescents, and young adults identified various individual and environmental factors that may offer significant protection [28]. At the individual level, self-esteem, emotional intelligence, and specific coping abilities were identified as key protective factors. Similarly, self-esteem, life satisfaction [29] and positive self-perceptions and less emotion-orientated coping were also identified as protective factors for SI in adolescence [30]. Nielassoff et al. [28] further emphasized the role of high-quality relationships with family and within the school environment as the most common protective factors for SA, while positive connections with peers, other adults, and cultural ties were also noted as protective. The importance of multi-dimensional ecological protective factors (social and environmental) for SI has also been emphasized by Gallagher et al. [23]. Nevertheless, the role of social protective factors for suicidality remains complex and, in some cases, puzzling. Although high levels of family attachment have been found to lower risk of SA [31] and SI [32, 33], peer relationships during adolescence have been reported to have mixed effects on SA and SI, depending on positive or negative influence [34]. Beyond immediate social circles, collective efficacy and community safety have been shown to significantly reduce adolescent SA [31]. Schools also play a crucial role in adolescents’ environmental context, with strong school connectedness, characterized by social support, belonging, and academic encouragement, linked to a reduced likelihood of SI and SA [35]. Socio-economic status (SES) is linked to several mental health outcomes, including suicidality [36]. Although the research on the direct effect of SES on suicidality is limited, a recent study found that adolescents with middle to middle-high SES had reduced odds of SI and SA compared to those with low or high SES [37].

While gender/sex differences are well established in suicidality [15, 19] and among specific risk factors [17], research on sex differences in protective factors for suicidality remains limited. Emerging evidence highlights the significance of this area of study. Higher levels of physical activity have been linked to reduced SA [28, 38] and SI [30]. However, a cross-sectional study of adolescents from 48 countries found that while high physical activity levels were associated with lower odds of SA in males, they were linked to higher odds in females [39]. Another cross-sectional study investigated the moderating role protective factors moderate the impact of stress on depression and suicidal behavior among adolescents in a community and clinical setting [40]. Results indicated that sex differences emerged in the predictive power of risk and protective factors, where positive focus (coping style) was a more effective protective factor for females, whereas self-discovery (spirituality) played a greater protective role for males. A population-based study found that attachment to parents and family functioning was significantly associated with reduced levels of SI among adolescent females, but not males [30]. Gaining a deeper understanding of sex-specific protective factors is essential for tailoring suicide prevention strategies in adolescents [23, 41].

### Rationale, objectives, and research questions of the current study

The rationale for this study was to expand the knowledge on protective factors for adolescent suicidality, an area where existing research remains limited and fragmented. While some studies distinguish between SI and SA, others do not, and inconsistencies in study design, age groups, and sample populations complicate comparisons. As highlighted by literature reviews and meta-analysis, most research on protective factors are cross-sectional, with few longitudinal studies, despite the need for such designs to capture the evolving nature of suicidality during adolescence. Gender/sex differences in suicidality are established, but potential differences in protective factors remain underexplored. Additionally, extensive research has been focused on high-risk subgroups, limiting the generalizability of findings to universal prevention. Investigating protective factors as independent variables, rather than mere moderators of risk, could help identify those that are universally beneficial, regardless of specific risk factors.

This study therefore aims to investigate the associations between protective factors in early adolescence and the subsequent outcomes of suicidal ideation (SI) and suicide attempts (SA) one year later, while also examining sex differences in these associations. The research specifically seeks to answer: (1) What protective factors in early adolescence are associated with a reduced likelihood of suicidal ideation and/or suicide attempts after one year? (2) How do these protective factors vary by sex?

## Method

### Study design and participants

Data stems from the “Youth and Mental Health Study” (YAMHS) undertaken in Norway. The YAMHS focused on adolescent depressive symptoms and conditions, but also explored risk and protective factors [42]. It commenced with its first data collection in 1998 (T1). Participants were recruited from two counties in central Norway. A representative sample of the population was selected, consisting of 2,813 students from 22 schools. The probability of selection was proportional to the size of each school (proportional allocation). Adolescents completed 2-hour self-report questionnaires during school hours. Full details of SES and other aspects of YAMHS main study have been described elsewhere [42]. Since T1 (1998), the YAMHS cohort has been followed up in three additional waves: T2 (1999, adolescence), T3 (2005, clinical subsample), and T4 (2012, young adulthood). This study utilized longitudinal data from the two adolescent timepoints, T1 and T2. The T1 sample comprised of 2,464 adolescents (response rate 88.3%, 1251 females and 1213 males, mean age 13.7 years (SD = 0.6)). Of those who participated at T1, 105 (4.3%) did not participate one year later (T2). Additionally, 73 new adolescents joined the study at T2. At T2 there were *N* = 2432 participants (1225 females and 1207 males), with a response rate of 87.1%, including with a mean age of 14.9 years (SD = 0.6). Reasons for non-participation at T2 included relocation, illness, refusal, serious disability, and leaving school. Non-participants were more likely to have higher levels of depressive symptoms and SI at T1 and a non-Norwegian background [42].

### Measures

#### Suicidal ideation (SI)

The outcome variable of SI at T2 was investigated using a mean score from five items: four items from the Mood and Feelings Questionnaire (MFQ) and one item from the Center for Epidemiological Studies Depression Scale (CES-D). This five-item composite scale has shown good reliability and been utilized in the YAMHS previously [43, 44]. MFQ assesses depressive symptoms among adolescents [45], including the four items that have been validated in detection of current and predictive SI [46] The MFQ-SI items were “I thought that life was not worth living”, “I thought about death or dying”, “I thought my family would be better off without me”, and “I thought about killing myself”. The additional item from the CES-D scale was added to obtain a more comprehensive embodiment of SI, that can also include more passive thoughts [43]. The CES-D item was “I would have killed myself if I had known a way of doing it” [47]. Responses to all five items were given as 0 = “not true”, 1 = “sometimes true or 2 = “true”. As the distribution of SI was highly skewed, internal consistency of the SI scale was computed using McDonalds Omega [48]. The Omega was 0.87, implying good internal consistency.

#### Suicide attempts (SA)

The outcome of SA at T2 was investigated using the single question “Have you ever attempted suicide?” as the dependent variable. This was one of four items in the YAMHS questionnaire used to assess self-harm and SA among adolescents and adults. The SA question and accompanying questions originated from the “Young in Norway” study [49] however, these were not part of any scale. There were three possible responses to the SA question 0: *No*, 1: *Yes*,* once*, and 2: *Yes*,* several times*. Prior to responding to the SA question there was an entry question “Have you ever on purpose taken an overdose of pills or in other ways tried to harm yourself?”, with the same response options. If participants had answered *no*, they were prompted to skip the SA question. For these participants, we imputed “No” in the SA question.

#### Protective factors

The protective factors examined in this study were selected based on prior research [28–32, 38] and theoretical frameworks [27] emphasizing both individual and ecological influences [23, 26, 41] that were also available in the YAMHS dataset. We focused on three main domains of protective factors: Individual, social and environmental. Individual protective factors include personal characteristics such as self-worth, coping and level of physical activity, while social and environmental factors encompass aspects such as family functioning, and school connectedness/wellbeing. These domains were chosen to reflect the multi-dimensional nature of protective influences on adolescent suicidality.

##### Individual factors

***Self-perception*** was measured by using the “Self-Perception Profile for Adolescents” (SPPA) [50]. The Norwegian version condensed the original nine subscales into seven following cultural adaptations and removal of scales with low reliability [51]. The YAMHS used three subscales of the SPPA, making 15 questions in total: “Physical Appearance”, “Social Competence” and “Global self-worth”. The physical appearance scale measures individuals’ perceptions of their physical appearance, body image, and perceived attractiveness. The social competence scale evaluates perceptions of peer popularity, friendship skills, and acceptance by peers. The global self-worth scale assesses an individual’s overall sense of self-worth or self-esteem. Responses were given on a four-point Likert scale (1 to 4), with higher scores indicating positive self-perceptions. The scales had good internal consistency, with McDonald’s Omega; 0.89 for Physical Appearance, 0.76 for Social Competence, and 0.80 for Global Self-Worth.

***Task-oriented coping*** traits were measured by a subscale from 1990 version of the Coping Inventory for Stressful Situations (CISS). The CISS scale measures theory-based coping dimensions and is known for its robust psychometric properties [52, 53]. Task-orientated coping is often associated with adaptive ways of managing stress, resulting in more positive outcomes. It involves actively facing stressful situations through a problem-focused approach, using strategies such as reframing, planning and seeking resources. The task-orientated coping subscale consisted of five 5 items, and responses were given on a four-point Likert Scale, where higher scores equate to more of the coping trait. The task-oriented coping subscale had good internal consistency, with McDonald’s Omega; 0.84.

The participants’ ***physical activity*** was measured using a single item, modified by Paffenbarger et al. [54]. and used in the YAMHS questionnaire. The item requested participants to rank their level of activity (physically inactive-highly physically active) using a five-point Likert scale. Higher scores suggest greater levels of physical activity.

##### Social factors

***Attachment*** was assessed using the revised version of the “Inventory of Parent and Peer Attachment” (IPPA), which includes separate scales for attachment to mother, father, and peers [55]. The IPPA evaluates adolescents’ perceptions of the cognitive and affective aspects of their relationships, with responses scored on a five-point Likert scale. The parent attachment scales each contain 25 items, while the peer attachment scale was shortened to 9 items in the YAMHS after removing low-reliability items from previous research, resulting in a unidimensional scale. Higher scores indicate more secure attachment. The IPPA demonstrates strong psychometric properties and good reliability [56, 57]. In this study, total mean scores were calculated separately for each of the three scales. Internal consistency was high for attachment to parents, with McDonald’s Omega values of 0.89 for attachment to mother and 0.88 for attachment to father. However, the attachment to peers scale showed lower internal consistency, with an McDonald’s Omega value of 0.75.

Adolescent perceptions of their ***family functioning*** were assessed using the “General Functioning” subscale from the McMaster Family Assessment Device (FAD) [58]. This subscale consists of 12 items, with responses recorded on a five-point Likert scale. Higher scores reflect better perceived family functioning as reported by the adolescents. The General Family Functioning scale demonstrated good internal consistency, with a McDonald’s Omega of 0.86.

##### Environmental factors

To investigate ***school connectedness and wellbeing***, five of the six-item scale developed for the longitudinal study “Young in Norway” called “Trivsel i Klassen” (translation: “school-class connectedness/wellbeing”) were used [59]. This scale is designed to measure students’ wellbeing within their school class environment, focusing on factors such as social support from peers, a sense of belonging, and general satisfaction with the classroom atmosphere. One item was excluded from the scale to increase the internal reliability of the scale. Responses were given on a four-point Likert scale (1 to 4), with higher scores indicating better school-class connectedness/wellbeing. The McDonald’s Omega for the scale was 0.72.

***Socio-Economic Status (SES)*** was calculated based on adolescent self-report on their parents’ occupational statuses. There were five response categories as classified by ISCO-88; manual workers, primary industry, lower middle class, upper middle class and professional leader [60], with higher scores suggesting higher SES.

### Statistical analysis

The internal consistency of scales was analyzed using McDonald’s Omega ω [61]. Mean score indices were computed when at least 80% of the items of the scales were completed. The proportion of missing data was less than 10% for each protective factor variable. Missing data were handled using available case analysis, meaning that each analysis included only participants with data on the variables under consideration. We compared the SI and SA between sexes using the Wilcoxon Mann-Whitney test. We used linear regression analyses with SI as dependent variable, and ordinal logistic regression analyses with SA as dependent variable. Since SI is heavily skewed, we used the robust variance estimator in the linear regression. Each analysis included one protective factor, sex, and their interaction as covariates. In these analyses, the effect of the protective factor for males are obtained with sex coded as zero for males, and respectively for females with sex coded zero for females. The Benjamini-Hochberg procedure was applied to adjust the 66 *p*-values for multiple testing, controlling the false discovery rate (FDR) across the analyses. Adjusted *p*-values below *p* <.05 were considered statistically significant, and 95% confidence intervals (CI) were reported where relevant. All analyses were conducted using Stata 18, with the Benjamini-Hochberg adjustment performed in R Studio.

## Results

### Descriptive characteristics

The distribution of the dependent variables suicide attempts ordinal scores (0, 1, and 2) by suicidal ideation scale (0–2) for the total sample is presented in Table 1. Additionally, detailed information for males and females is provided in separate tables in the supplementary materials (Additional file 1).

**Table 1:** Distribution of the suicidal ideation scale mean scores (0–2) by suicide attempts ordinal scores (0, 1, and 2) for the total sample

Suicidal ideation scale, mean scores (0–2)	Suicide attempts ordinal scores (0,1 and 2)			Total
	No	Yes, once	Yes, more than once	
0.00	1768	21	2	1791
0.20–0.39	246	11	4	261
0.40–0.49	88	8	3	99
0.60–0.79	57	6	4	67
0.80–0.99	36	10	4	50
1.00–1.19	18	7	5	30
1.20–1.39	13	2	5	20
1.40–1.59	11	5	1	17
1.60–2.00	13	16	15	44
Total	2250	86	43	2379

Note. Data reported at T2

Females reported higher levels of SI (M = 0.19, SD = 0.39) than males (M = 0.09, SD = 0.27), and this difference was highly significant (*p* <.001). Among adolescent females, 2.1% (*n* = 25) reported more than one SA, 5.4% (*n* = 65) reported one SA, and 92.5% (*n* = 1,107) reported no SA. Among adolescent males, 1.5% (*n* = 18) reported more than one SA, 1.8% (*n* = 21) reported one SA, and 96.7% (*n* = 1,145) reported no SA. These differences between the sexes in SA were also highly significant (*p* <.001). Descriptive statistics for the protective factors are presented in Table 2.

**Table 2:** Descriptive statistics for the protective factors

Measure	Scale	Sex	*n*	Min	Max	Mean	SD
**Individual factors**							
Self-perception							
Physical appearance	1–4	F	1187	1.00	4.00	2.48	0.73
		M	1171	1.00	4.00	3.03	0.67
Social competence	1–4	F	1192	1.00	4.00	3.11	0.52
		M	1178	1.00	4.00	3.22	0.52
Global self-worth	1–4	F	1192	1.00	4.00	2.85	0.62
		M	1180	1.00	4.00	3.21	0.57
Task-oriented coping	1–4	F	1179	1.00	4.00	2.54	0.65
		M	1159	1.00	4.00	2.53	0.61
Physical activity	1–5	F	1232	1.00	5.00	3.64	1.24
		M	1187	1.00	5.00	3.93	1.23
***Social factors***							
Attachment to mother	1–5	F	1178	1.80	4.84	3.88	0.53
		M	1148	1.52	4.84	3.87	0.52
Attachment to father	1–5	F	1129	1.48	4.84	3.74	0.56
		M	1113	1.32	4.84	3.72	0.58
Attachment to peers	1–5	F	1216	1.00	5.00	3.17	0.59
		M	1185	1.00	5.00	3.20	0.56
Family functioning	1–4	F	1163	1.00	4.00	3.18	0.50
		M	1129	1.00	4.00	3.72	0.48
***Environmental factors***							
School connectedness/wellbeing	1–4	F	1232	1.00	4.00	3.19	0.53
		M	1193	1.00	4.00	3.23	0.59
Socio-economic status	1–5	F	1205	1.00	5.00	2.67	1.50
		M	1169	1.00	5.00	2.64	1.45

Note. F = females, M = males

### Associations between protective factors (T1) and suicidal ideation and suicide attempts (T2) in adolescents

There were statistically significant associations between self-perceptions (specifically physical appearance, social competence, and global self-worth) in early adolescence (T1) and self-reported SI (Table 3) and SA (Table 4) one year later (T2) for both females and males. A significant sex difference (*p* =.015) was observed in the relationship between self-worth and SI, with females showing a stronger association (*B* = -0.16, CI [-0.20 to -0.12], *p* <.001) compared with males (*B* = -0.08, CI [-0.12 to -0.04], *p* <.001).

Social competence was associated with lower odds of SA for males (OR = 0.24, CI [0.13 to 0.42], *p* <.001) compared to females (OR = 0.53, CI [0.35 to 0.80], *p* =.009). The adjusted *p*-value for the interaction term was 0.083, suggesting that the sex difference in this effect showed a tendance towards significance.

Higher levels of physical activity was significantly associated with lower levels SI in both females (*B* = -0.04, CI [-0.06 to -0.02], *p* <.001) and males (*B* = -0.02, CI [-0.04 to -0.01], *p* =.009), with no significant sex differences in this association (*p* =.570).

Additionally, higher levels of school connectedness/wellbeing in early adolescence were significantly associated with both lower levels of SI and SA one year later. For SI, higher school connectedness/wellbeing was associated with decreased levels of SI for females (*B* = -0.14, CI [-0.19 to -0.08], *p* <.001) and males (*B* = -0.09, CI [-0.13 to -0.05], *p* <.001), with no significant sex differences observed (*p* =.392). Similarly, for SA, higher school connectedness/wellbeing was associated with a decreased odds of SA for females (OR = 0.46, CI [0.32 to 0.66], *p* <.001) and males (OR = 0.31, CI [0.18 to 0.51], *p* <.001), with no significant sex differences in this effect (*p* =.448).

There were no statistically significant associations between task-oriented coping, attachment (to mother, father, or peers), family functioning or SES and SI or SA one year later, either for females or males (Tables 3 and 4).

**Table 3:** Linear regression with suicidal ideation at time 2 as dependent variable, and the protective factor at time 1, sex and their interaction as covariates

Suicidal ideation	Females					Males					
			95% CI					95% CI			
	*n*	*B*	*LL*	*UL*	*p-*value	*n*	*B*	*LL*	*UL*	*p-*value	*Intx.* ^*a*^
***Individual factors***											
Self-perception											
Physical appearance	1139	-0.10	-0.13	-0.07	**< 0.001**	1127	-0.06	-0.09	-0.03	**< 0.001**	0.249
Social competence	1143	-0.10	-0.15	-0.04	**0.001**	1133	-0.08	-0.12	-0.04	**< 0.001**	0.983
Global self-worth	1142	-0.16	-0.20	-0.12	**< 0.001**	1135	-0.08	-0.12	-0.04	**< 0.001**	**0.015**
Task-oriented coping	1123	0.02	-0.02	0.06	0.651	1117	-0.01	-0.03	0.01	0.651	0.458
Physical Activity	1177	-0.04	-0.06	-0.02	**< 0.001**	1141	-0.02	-0.04	-0.01	**0.009**	0.570
***Social factors***											
Attachment to mother	1123	0.01	-0.03	0.04	0.903	1008	-0.01	-0.04	0.02	0.792	0.792
Attachment to father	1078	0.00	-0.04	0.04	0.942	1071	-0.01	-0.03	0.02	0.903	0.911
Attachment to peers	1160	0.01	-0.03	0.04	0.911	1142	0.00	-0.03	0.03	0.918	0.903
Family functioning	1111	-0.01	-0.05	0.04	0.918	1088	-0.01	-0.04	0.02	0.813	0.942
***Environmental factors***											
School connectedness/ wellbeing	1175	-0.14	-0.19	-0.08	**< 0.001**	1146	-0.09	-0.13	-0.05	**< 0.001**	0.448
Socio-economic status	1148	0.01	-0.01	0.02	0.868	1124	-0.01	-0.02	0.00	0.444	0.502

*Note. p*-values are adjusted using the Benjamini-Hochberg procedure

^a^*p*-value for the interaction sex*protective factor

**Table 4:** Ordinal logistic regression with suicide attempts at time 2 as dependent variable, and the protective factor at time 1, sex and their interaction as covariates

Suicide Attempts	Females					Males					
			95% CI					95% CI			
	*n*	*OR*	*LL*	*UL*	*p-*value	*n*	*OR*	*LL*	*UL*	*p-*value	*Intx.* ^a^
***Individual factors***											
Self-perception											
Physical appearance	1119	0.47	0.35	1.54	**< 0.001**	1113	0.43	0.28	0.69	**< 0.001**	0.903
Social competence	1123	0.53	0.35	0.80	**0.009**	1118	0.24	0.13	0.42	**< 0.001**	**0.083**
Global self-worth	1122	0.33	0.23	0.47	**< 0.001**	1120	0.34	0.19	0.57	**< 0.001**	0.987
Task-oriented coping	1099	1.13	0.80	1.59	0.792	1101	0.73	0.43	1.24	0.524	0.448
Physical Activity	1155	0.83	0.70	0.99	0.111	1125	0.78	0.61	0.01	0.178	0.903
***Social factors***											
Attachment to mother	1098	1.17	0.75	1.82	0.792	1092	0.81	0.44	2.49	0.792	0.651
Attachment to father	1055	1.04	0.69	1.60	0.918	1055	1.05	0.69	1.60	0.981	0.942
Attachment to peers	1135	1.13	0.77	1.65	0.826	1125	0.96	0.54	1.74	0.942	0.903
Family functioning	1079	0.96	0.61	0.51	0.918	1044	0.85	0.43	1.68	0.893	0.911
***Environmental factors***											
School connectedness/ wellbeing	1154	0.46	0.32	0.66	**< 0.001**	1129	0.31	0.18	0.51	**< 0.001**	0.457
Socio-economic status	1124	0.95	0.82	1.10	0.792	1108	0.86	0.67	1.09	0.472	0.792

Note. *p*-values are adjusted using the Benjamini-Hochberg procedure

^a^*p*-value for the interaction sex*protective factor

## Discussion

This study investigated the associations between protective factors and suicidal ideation (SI) and suicide attempts (SA) during early adolescence, examining potential sex differences. Results revealed significant associations between self-perceptions (including physical appearance, social competence, and global self-worth) and both SI and SA one year later for both sexes. Notably, females exhibited a stronger link between global self-worth and SI compared to boys. The interaction between sex and social competence significantly affected the likelihood of SA, with males showing a greater reduction than females, though this effect only showed a trend towards significance after adjustment. Higher levels of physical activity and school connectedness/wellbeing were associated with lower levels of SI in both sexes, with no significant differences between sexes. Additionally, school connectedness/wellbeing was linked to lower levels of SA in both sexes, with no significant sex differences. However, no significant associations were observed between task-oriented coping, attachment, family functioning or SES and SI or SA.

### The prominent role of self-perceptions as a protective factor for suicidality in adolescence

In line with existing literature [28–30], positive self-perceptions, were significantly associated with a reduction in SI and SA across sexes. However, the association between global self-worth and SI was stronger in females compared to males. Additionally, males showed a greater reduction in SA when levels of social competence increased, compared to females. High self-worth is associated with greater psychological resilience, enabling individuals to better cope with stress, adversity, and emotional challenges [62]. When individuals have a positive view of own worth and abilities, they may be better equipped to perceive life’s difficulties as manageable, potentially decreasing the likelihood of suicidality. Additionally, higher self-worth is often linked to stronger social connections, as these individuals typically cultivate positive and supportive relationships [63]. These enhanced social connections may contribute to the protective effect against suicidality.

The stronger association between global self-worth and SI in females compared to males may be explained by sex differences in how self-worth/esteem is influenced by social and emotional factors. Previous studies suggest that adolescent females tend to be more emotionally responsive to interpersonal stresses and social acceptance [64], which makes their overall self-worth particularly sensitive to factors such as social rejection, body image concerns, and relational conflicts. For males, the greater reduction in odds of SA associated with higher levels of social competence could be related to societal expectations around masculinity and emotional expression [65]. Males may place a higher value on social competence, such as peer interactions, social dominance, and problem-solving abilities, which can enhance their sense of agency and control [66]. These traits are often seen as markers of success in social settings, and improvements in social competence might reduce feelings of isolation or helplessness, leading to reduced likelihood of SA. Since adolescent males are less likely to seek help for mental health problems and suicidality [67], increased social competence could also equip them with alternative coping mechanisms, reducing the likelihood of acting on suicidal thoughts.

### Physical activity and school connectedness/wellbeing

In our study, we found that physical activity was significantly associated with a reduction in SI over a one-year period. However, the association between physical activity and the odds of SA was not statistically significant, despite a slight reduction in odds being observed. This discrepancy between SI and SA may be attributed to several factors, including limited statistical power due to the relatively low number of reported SA within the study sample. This finding aligns with existing research highlighting the positive impact of physical activity on suicidality [28, 30, 38]. Engaging in regular exercise promotes emotional regulation, improves self-esteem, and provides opportunities for social interaction [68], all of which may help reduce negative mental health outcomes and thoughts of suicide. It is also important to recognize that SI and SA are distinct phenomena, and physical activity may have differential effects on these outcomes. SI represents thoughts that may involve wishes and contemplations of suicide, which may be more responsive to mood-altering effects of physical activity. On the other hand, SA involve a transition from thought to action [69] which may be influenced by additional factors such as impulsivity, access to means, and acute stressors, potentially making them less directly modifiable by factors like physical activity alone. In contrast, positive self-perceptions predicted a reduction in SA one year later, suggesting a more sustained and direct impact. Future research with larger sample sizes and more frequent assessments may clarify whether physical activity can significantly reduce SA.

Consistent with earlier studies [28, 30, 35], our findings indicate that positive school environments are significantly protective, also over the course of one year for both SI and SA, with no significant sex difference. These results emphasize that the school plays a central role in adolescents’ connectedness and wellbeing, encompassing social integration and emotional support. Education and school environments, in combination with other social and environmental factors such as community support and access to resources, are essential in fostering resilience, which significantly influences long-term mental health outcomes, as outlined by the World Health Organization and Calouste Gulbenkian Foundation [70]. This underscores the importance of social determinants in promoting mental well-being throughout life. These dimensions may contribute to a positive and nurturing social environment that appears to provide adolescents with valuable resources for coping with stress and fostering a sense of belonging, with an effect of reducing SI and SA. As the protective effects observed were consistent across sexes, this indicates that benefits of school connectedness/wellbeing may be universally applicable to adolescents, regardless of presence of risks. Previous studies are limited by cross-sectional nature [35], and findings of this study suggest that the school environment also has a protective effect over time. Results highlight the importance of schools in creating a supportive atmosphere in prevention of suicidality, although further research is needed to confirm these findings and explore the mechanisms through which school environments impact adolescent mental health.

### No significant longitudinal association between parental/peer attachment, family functioning, task-oriented coping, SES and SI or SA over one year

In contrast to previous findings [28, 31, 32], we found no significant association between attachment to parents or peers and family functioning with SI or SA one year later. One possible explanation for the discrepancy between our findings and those of previous cross-sectional studies is the difference in study design. Cross-sectional studies provide a snapshot of the relationship between attachment, family functioning, and suicidality but do not capture changes over time. It is possible that over a year, the immediate effects of insecure attachment or family dysfunction might diminish as other factors, such as changes in mental health status, become more influential. Moreover, it is important to consider that attachment to parents and family functioning may have indirect effects on adolescent suicidality through other variables, such as social competence, self-esteem, or mental health symptoms. For instance, insecure attachment might exacerbate depressive symptoms or impair self-worth, both of which are linked to suicidality. Additionally, cognitive, emotional, and social development during adolescence can alter relationships with parents, peers, and family, potentially reducing the lasting impact of attachment and family functioning on SI. As adolescents become more independent, they may rely less on family support and more on peers or external resources [71]. Supportive school environments and physical activities may help manage emotional difficulties, thereby lessening the long-term impact of insecure attachment or family dysfunction on suicidality. Future research should explore these factors together and use multivariate analyses to better understand their combined effects on adolescent suicidality.

We found no significant association between task-oriented coping or SES and SI and SA. While coping strategies and SES are recognized as important variables in suicidality research, their inclusion as protective factors remain complex. For instance, although low SES is associated with a greater risk of suicidality [36], evidence regarding higher SES as a protective factor is inconclusive. Some studies suggest that higher SES might, under specific conditions, such as heightened academic or social pressures, increase risk [72]. In the context of suicidality, middle to middle-high SES may be more protective, than low or high SES [37]. Similarly, task-oriented coping can potentially become maladaptive rather than protective if the stressor is highly emotive, harder to problem-solve and is perceived as less controllable [73], which may reflect stressors associated with suicidality. While task-oriented coping may promote resilience in some individuals, its effectiveness likely also depends on the availability of external support and individual differences in coping flexibility. These findings underscore the nuanced and context-dependent roles of SES and coping strategies in suicidality, highlighting the need for further research to clarify their mechanisms as protective factors.

### Strengths and limitations of the study

The study’s use of a large, representative population sample is a notable strength, complemented by very low attrition from Time 1 to Time 2 (4.3%). However, the participants who did not continue at T2 had significantly higher levels of depressive symptoms, including SI at T1 [42]. This may result in a slight underestimation of the associations between protective factors and suicidality, as those at higher risk were less likely to be included in the follow-up. The incorporation of sex stratification, an aspect often overlooked in previous research on protective factors for suicidality, allowed for the identification of sex-specific protective factors that might be missed in aggregated analyses. This stratification revealed variations in the strength and direction of associations, providing nuanced insights into sex-specific influences on suicidality. Additionally, protective factors were selected based on established research and theoretical frameworks. By focusing on main effects rather than mediators or moderators, the study identified factors that are universally beneficial, enhancing the relevance and applicability of the findings across diverse contexts.

Several limitations must be noted when interpreting findings of this study. The one-year follow-up period may be insufficient to capture long-term effects or changes in suicidality among adolescents, suggesting that longer follow-up periods could provide a more comprehensive understanding of these dynamics. Although the sample size is large, subgroup analyses, such as those by sex or specific protective factors and SA, might suffer from limited statistical power, affecting the robustness of findings for smaller subgroups. The reliance on self-reported data for SI and SA introduces potential biases, such as underreporting or overreporting, which can distort the true prevalence [74]. The present study only explored binary sex, as self-report of other non-binary gender groups was not obtained from participants. Integrating self-report measures with external data sources, such as medical records or registry data, could supplement the self-reported assessment of suicide risk and protective factors. Furthermore, while the study’s sample is representative of the population in Norway, it has limited ethnic diversity [42] which may affect the generalizability of the findings. Several protective factors not investigated in this study, such as coping abilities [28], cultural influences [75] religiosity/spirituality and positive diversion activities (i.e., reading books, watching movies) [76], remain partially explored and warrant further investigation. Finally, the interplay between protective factors, such as the relationship between positive self-perceptions and social relationships, requires further exploration.

### Implications and future directions

This study highlights the crucial role of positive self-perception in preventing suicidality (both SI and SA) during early adolescence, while also revealing sex-specific nuances in these associations. Improving adolescent self-perceptions of global self-worth, physical appearance and social competence, is essential, especially in today’s social media-driven environment that may negatively affect mental health [77]. Although evidence for self-perception/esteem enhancing interventions are limited, a recent systematic review and meta-analysis identified significant effects in reducing suicidality, especially SI [78]. Targeting self-esteem, which is integral to self-perceived worth and competencies, can potentially reduce suicidality among females and males according to findings of our study. Universal school-based programs like “Youth Aware of Mental Health” offer valuable strategies for mental health promotion that increases mental health literacy, reduces stigma and has proven effectiveness in reducing suicidality among adolescents [79, 80]. Efforts of early prevention programs delivered in school-settings may in the process also foster more accepting and supportive school environments, while contributing to improved self-perceptions. Furthermore, existing programs and interventions benefit from incorporating physical activity due to the evident positive impact on adolescent mental health and wellbeing [81], including possibly reducing SI as identified in this study. Despite a general focus on minimizing risk factors, there is a pressing need to promote protective factors in universal prevention strategies. Given that SI is a precursor for SA, and that both are associated with long-term negative consequences [9, 10], early prevention during adolescence can significantly reduce present and future suicidality. Although family functioning and attachment to parents and peers were not identified as primary factors in reducing suicidality in the present study, they may have indirect protective impact. Hence, acknowledging and incorporating resources of family and peers remain important in the frameworks of interventions targeting suicide prevention. Moreover, it is important to note that the effectiveness of the above protective factors varies by individual circumstances and does not guarantee immunity from suicidality. Future research should also further explore protective factors across sex and gender groups. Tailoring interventions to address both universal and individual vulnerabilities identified during adolescence, while simultaneously enhancing protective factors, is essential for effective practice. By enhancing understanding and leveraging protective factors, we can develop targeted prevention strategies that build on strengths, resilience, and hope, ultimately empowering individuals and communities to reduce suicidality.

## Conclusion

Positive self-perceptions, encompassing physical appearance, social competence, and global self-worth, are significantly associated with reduced levels of both SI and SA one year later. Notably, self-worth is more strongly associated with reduced SI in females compared to males. Additionally, social competence is associated with lower odds of SA in males relative to females, although this association only trends towards significance. Higher levels of physical activity and school connectedness/wellbeing were linked to lower levels of SI across both sexes, with no significant sex differences observed. Increased school connectedness/wellbeing were associated with lower levels of SA across both sexes. Task-oriented coping, attachment, family functioning and SES did not demonstrate significant associations with SI or SA over the one-year period. Conclusively, these results highlight the importance of focusing on self-perception, physical activity and school connectedness/wellbeing in preventative strategies, while underscoring the need for sex-sensitive approaches.

## Electronic supplementary material

Below is the link to the electronic supplementary material.

Supplementary Material 1

## Data Availability

Datasets used in this study, from the Youth and Mental Health Study (YAMHS) are not readily available to the public and is restricted by the Regional Committees for Medical and Health Research Ethics. Data may be made available upon appropriate request to the Norwegian University of Science and Technology (NTNU), Department of Mental Health, Regional Centre for Child and Youth Mental Health and Child Welfare.

## References

[1] Masquelier B, Hug L, Sharrow D, You D, Mathers C, Gerland P, et al. Global, regional, and national mortality trends in youth aged 15–24 years between 1990 and 2019: a systematic analysis. Lancet Global Health. 2021;9(4):e409–17. 33662320 10.1016/S2214-109X(21)00023-1 PMC7966666

[2] VandenBos GR. APA dictionary of psychology. American Psychological Association; 2007.

[3] Harmer B, Lee S, Duong TVH, Saadabadi A. Suicidal Ideation Treasure Island (FL): StatPearls Publishing LLC;, Harmer B, Lee S, Rizvi A et al. Suicidal Ideation. [Updated 2024 Apr 20]. In: StatPearls [Internet]. Treasure Island (FL): StatPearls Publishing; 2024 Jan-. Available from: https://www.ncbi.nlm.nih.gov/books/NBK565877/

[4] Bilsen J. Suicide and youth: risk factors. Front Psychiatry. 2018;9:540. 30425663 10.3389/fpsyt.2018.00540 PMC6218408

[5] Turecki G, Brent DA. Suicide and suicidal behaviour. Lancet. 2016;387(10024):1227–39. 26385066 10.1016/S0140-6736(15)00234-2 PMC5319859

[6] Van Meter AR, Knowles EA, Mintz EH. Systematic review and meta-analysis: international prevalence of suicidal ideation and attempt in youth. J Am Acad Child Adolesc Psychiatry. 2023;62(9):973–86. 36563876 10.1016/j.jaac.2022.07.867 PMC12702477

[7] Mars B, Heron J, Klonsky ED, Moran P, O’Connor RC, Tilling K, et al. Predictors of future suicide attempt among adolescents with suicidal thoughts or non-suicidal self-harm: a population-based birth cohort study. Lancet Psychiatry. 2019;6(4):327–37. 30879972 10.1016/S2215-0366(19)30030-6 PMC6494973

[8] Rossom RC, Coleman KJ, Ahmedani BK, Beck A, Johnson E, Oliver M, et al. Suicidal ideation reported on the PHQ9 and risk of suicidal behavior across age groups. J Affect Disord. 2017;215:77–84. 28319695 10.1016/j.jad.2017.03.037 PMC5412508

[9] Paashaus L, Forkmann T, Glaesmer H, Juckel G, Rath D, Schönfelder A, et al. From decision to action: suicidal history and time between decision to die and actual suicide attempt. Clin Psychol Psychother. 2021;28(6):1427–34. 33687121 10.1002/cpp.2580

[10] Miche M, Studerus E, Meyer AH, Gloster AT, Beesdo-Baum K, Wittchen H-U, et al. Prospective prediction of suicide attempts in community adolescents and young adults, using regression methods and machine learning. J Affect Disord. 2020;265:570–8. 31786028 10.1016/j.jad.2019.11.093

[11] Blakemore S-J, Mills KL. Is adolescence a sensitive period for sociocultural processing? Ann Rev Psychol. 2014;65(1):187–207. 24016274 10.1146/annurev-psych-010213-115202

[12] Steinberg L. Cognitive and affective development in adolescence. Trends Cogn Sci. 2005;9(2):69–74. 15668099 10.1016/j.tics.2004.12.005

[13] Patton GC, Sawyer SM, Santelli JS, Ross DA, Afifi R, Allen NB, et al. Our future: a Lancet commission on adolescent health and wellbeing. Lancet. 2016;387(10036):2423–78. 27174304 10.1016/S0140-6736(16)00579-1 PMC5832967

[14] World Health Organization. Preventing suicide: a global imperative. World Health Organization; 2014. p. 9241564776. Report No.

[15] Canetto SS, Sakinofsky I. The gender paradox in suicide. Suicide Life-Threatening Behav. 1998;28(1):1–23. 9560163

[16] Adrian M, Miller AB, McCauley E, Vander Stoep A. Suicidal ideation in early to middle adolescence: sex-specific trajectories and predictors. J Child Psychol Psychiatry. 2016;57(5):645–53. 26610726 10.1111/jcpp.12484 PMC4837032

[17] Miranda-Mendizabal A, Castellví P, Parés-Badell O, Alayo I, Almenara J, Alonso I, et al. Gender differences in suicidal behavior in adolescents and young adults: systematic review and meta-analysis of longitudinal studies. Int J Public Health. 2019;64(2):265–83. 30635683 10.1007/s00038-018-1196-1 PMC6439147

[18] Cantor N, Kingsbury M, Warner E, Landry H, Clayborne Z, Islam R et al. Young adult outcomes associated with adolescent suicidality: a meta-analysis. Pediatrics. 2023;151(3). 10.1542/peds.2022-058113 36810672

[19] Roth G. Global Burden of Disease Collaborative Network. Global Burden of Disease Study 2017 (GBD 2017) Results. Seattle, United States: Institute for Health Metrics and Evaluation (IHME), 2018. The Lancet. 2018;392:1736-88.

[20] Millner AJ, Robinaugh DJ, Nock MK. Advancing the understanding of suicide: the need for formal theory and rigorous descriptive research. Trends Cogn Sci. 2020;24(9):704–16. 32680678 10.1016/j.tics.2020.06.007 PMC7429350

[21] Bryan CJ. Rethinking suicide: why prevention fails, and how we can do better. Oxford University Press; 2021.

[22] O’Connor RC, Nock MK. The psychology of suicidal behaviour. Lancet Psychiatry. 2014;1(1):73–85. 26360404 10.1016/S2215-0366(14)70222-6

[23] Gallagher ML, Miller AB. Suicidal thoughts and behavior in children and adolescents: an ecological model of resilience. Adolesc Res Rev. 2018;3:123–54. 29904718 10.1007/s40894-017-0066-z PMC5995470

[24] Stone DM, Holland KM, Bartholow BN, Crosby AE, Davis SP, Wilkins N. Preventing suicide: A technical package of policies, programs, and practice. 2017.

[25] Sher L. Resilience as a focus of suicide research and prevention. Acta Psychiatrica Scandinavica. 2019;140(2):169–80. 31150102 10.1111/acps.13059

[26] Bronfenbrenner U. Developmental ecology through space and time: A future perspective. 1995.

[27] Zimmerman MA. Resiliency theory: a strengths-based approach to research and practice for adolescent health. Sage Publications Sage CA: Los Angeles, CA;; 2013. pp. 381–3. 10.1177/1090198113493782 PMC3966565 23863911

[28] Nielassoff E, Le Floch M, Avril C, Gohier B, Duverger P, Riquin E. Protective factors of suicidal behaviors in children and adolescents/young adults: a literature review. Archives de Pédiatrie. 2023;30(8):607–16. 37777349 10.1016/j.arcped.2023.07.006

[29] Zhu X, Tian L, Huebner ES. Trajectories of suicidal ideation from Middle Childhood to Early Adolescence: risk and protective factors. J Youth Adolesc. 2019;48(9):1818–34. 31346925 10.1007/s10964-019-01087-y

[30] Bakken V, Lydersen S, Skokauskas N, Sund AM, Kaasbøll J. Protective factors for suicidal ideation -A prospective study from adolescence to adulthood. Eur Child Adolesc Psychiatry. 2024. 10.1007/s00787-024-02379-w PMC11424721 38356041

[31] Maimon D, Browning CR, Brooks-Gunn J. Collective efficacy, family attachment, and urban adolescent suicide attempts. J Health Soc Behav. 2010;51(3):307–24. 20943592 10.1177/0022146510377878 PMC3110665

[32] Yang H, Ran G, Zhang Q, Niu X. The Association between parental attachment and youth suicidal ideation: a three-level Meta-analysis. Archives Suicide Res. 2023;27(2):453–78. 10.1080/13811118.2021.2020192 34964432

[33] Guo Y, Ji Y, Huang Y, Jin M, Lin Y, Chen Y, et al. The relationship between suicidal ideation and parental attachment among adolescents: the mediator of anhedonia and peer attachment. Front Psychol. 2021;12:727088. 34733205 10.3389/fpsyg.2021.727088 PMC8558217

[34] Schlagbaum P, Tissue JL, Sheftall AH, Ruch DA, Ackerman JP, Bridge JA. The impact of peer influencing on adolescent suicidal ideation and suicide attempts. J Psychiatr Res. 2021;140:529–32. 34167026 10.1016/j.jpsychires.2021.06.027

[35] Marraccini ME, Brier ZM. School connectedness and suicidal thoughts and behaviors: a systematic meta-analysis. School Psychol Q. 2017;32(1):5. 10.1037/spq0000192 PMC5359058 28080099

[36] Madigan A, Daly M. Socioeconomic status and depressive symptoms and suicidality: the role of subjective social status. J Affect Disord. 2023;326:36–43. 36709827 10.1016/j.jad.2023.01.078

[37] Kim KM. What makes adolescents psychologically distressed? Life events as risk factors for depression and suicide. Eur Child Adolesc Psychiatry. 2021;30:359–67. 32232580 10.1007/s00787-020-01520-9

[38] Vancampfort D, Hallgren M, Firth J, Rosenbaum S, Schuch FB, Mugisha J, et al. Physical activity and suicidal ideation: a systematic review and meta-analysis. J Affect Disord. 2018;225:438–48. 28858658 10.1016/j.jad.2017.08.070

[39] Felez-Nobrega M, Haro JM, Vancampfort D, Koyanagi A. Sex difference in the association between physical activity and suicide attempts among adolescents from 48 countries: a global perspective. J Affect Disord. 2020;266:311–8. 32056893 10.1016/j.jad.2020.01.147

[40] Breton JJ, Labelle R, Berthiaume C, Royer C, St-Georges M, Ricard D, et al. Protective factors against depression and suicidal behaviour in adolescence. Can J Psychiatry. 2015;60(2 Suppl 1):S5–15. 25886672 PMC4345848

[41] McLean J, Maxwell M, Platt S, Harris FM, Jepson R. Risk and protective factors for suicide and suicidal behaviour: a literature review. Scottish Government; 2008.

[42] Kaasbøll J, Sigurdson JF, Skokauskas N, Sund AM. Cohort profile: the Youth and Mental Health Study (YAMHS) - a longitudinal study of the period from adolescence to adulthood. PLoS ONE. 2021;16(2):e0247036. 33606731 10.1371/journal.pone.0247036 PMC7895392

[43] Undheim AM. Involvement in bullying as predictor of suicidal ideation among 12-to 15-year-old Norwegian adolescents. Eur Child Adolesc Psychiatry. 2013;22(6):357–65. 23361192 10.1007/s00787-012-0373-7

[44] Sigurdson JF, Undheim AM, Wallander JL, Lydersen S, Sund AM. The Longitudinal Association of Being Bullied and gender with suicide Ideations, Self-Harm, and suicide attempts from adolescence to Young Adulthood: a Cohort Study. Suicide Life Threat Behav. 2018;48(2):169–82. 28581700 10.1111/sltb.12358

[45] Angold A. Structured assessments of psychopathology in children and adolescents. 1989.

[46] Hammerton G, Zammit S, Potter R, Thapar A, Collishaw S. Validation of a composite of suicide items from the Mood and feelings Questionnaire (MFQ) in offspring of recurrently depressed parents. Psychiatry Res. 2014;216(1):82–8. 24534124 10.1016/j.psychres.2014.01.040

[47] Andrews JA, Lewinsohn PM, Hops H, Roberts RE. Psychometric properties of scales for the Measurement of Psychosocial Variables Associated with Depression in Adolescence1. Psychol Rep. 1993;73(3part1):1019–46. 8302975 10.2466/pr0.1993.73.3.1019

[48] Kalkbrenner MT, Alpha. Omega, and H internal consistency reliability estimates: reviewing these options and when to use them. Couns Outcome Res Evaluation. 2021:1–12.

[49] Wichstrøm L. Predictors of adolescent suicide attempts: a nationally representative longitudinal study of Norwegian adolescents. J Am Acad Child Adolesc Psychiatry. 2000;39(5):603–10. 10802978 10.1097/00004583-200005000-00014

[50] Harter S. Self-perception profile for adolescents. Gifted Child Q. 1988.

[51] Wichstraum L. Harter’s Self-Perception Profile for adolescents: reliability, validity, and evaluation of the question format. J Pers Assess. 1995;65(1):100–16. 7643294 10.1207/s15327752jpa6501_8

[52] Endler NS, Parker JD. Assessment of multidimensional coping: Task, emotion, and avoidance strategies. Psychol Assess. 1994;6(1):50.

[53] Endler NS, Parker JDA, Ridder DTD, van Heck GL. Coping inventory for stressful situations: Multi-Health systems Incorporated; 1990.

[54] Paffenbarger RS Jr, Wing AL, Hyde RT. Physical activity as an index of heart attack risk in college alumni. Am J Epidemiol. 1978;108(3):161–75. 707484 10.1093/oxfordjournals.aje.a112608

[55] Armsden GC, Greenberg MT. The inventory of parent and peer attachment: individual differences and their relationship to psychological well-being in adolescence. J Youth Adolesc. 1987;16(5):427–54. 24277469 10.1007/BF02202939

[56] Gullone E, Robinson K. The inventory of parent and peer attachment—revised (IPPA-R) for children: a psychometric investigation. Clin Psychol Psychotherapy: Int J Theory Pract. 2005;12(1):67–79.

[57] Sund AM, Wichstrøm L. Insecure attachment as a risk factor for future depressive symptoms in early adolescence. J Am Acad Child Adolesc Psychiatry. 2002;41(12):1478–85. 12447035 10.1097/00004583-200212000-00020

[58] Epstein NB, Baldwin LM, Bishop DS. The McMaster family assessment device. J Marital Fam Ther. 1983;9(2):171–80.

[59] Wichstrøm L. Ung i Norge. Norsk Epidemiologi. 2002;12(3).

[60] Hoffmann E, Scott M. The revised International Standard Classification of Occupations (ISC0-88). Labour Statistics for a Market Economy: Challenges and Solutions in the Transition Countries of Central and Eastern Europe and the Former Soviet Union. 1995:189.

[61] Kalkbrenner MT. Alpha, omega, and H internal consistency reliability estimates: reviewing these options and when to use them. Couns Outcome Res Evaluation. 2023;14(1):77–88.

[62] Davey M, Eaker DG, Walters LH. Resilience processes in adolescents: personality profiles, self-worth, and coping. J Adolesc Res. 2003;18(4):347–62.

[63] DuBois DL, Burk-Braxton C, Swenson LP, Tevendale HD, Lockerd EM, Moran BL. Getting by with a little help from self and others: self-esteem and social support as resources during early adolescence. Dev Psychol. 2002;38(5):822. 12220058 10.1037//0012-1649.38.5.822

[64] Rudolph KD. Gender differences in emotional responses to interpersonal stress during adolescence. J Adolesc Health. 2002;30(4):3–13. 11943569 10.1016/s1054-139x(01)00383-4

[65] Mahalik JR, Burns SM, Syzdek M. Masculinity and perceived normative health behaviors as predictors of men’s health behaviors. Soc Sci Med. 2007;64(11):2201–9. 17383784 10.1016/j.socscimed.2007.02.035

[66] Smolak L, Stein JA. The relationship of drive for muscularity to sociocultural factors, self-esteem, physical attributes gender role, and social comparison in middle school boys. Body Image. 2006;3(2):121–9. 18089215 10.1016/j.bodyim.2006.03.002

[67] Calear AL, Morse AR, Batterham PJ, Forbes O, Banfield M. Silence is deadly: a controlled trial of a public health intervention to promote help-seeking in adolescent males. Suicide Life‐Threatening Behav. 2021;51(2):274–88. 10.1111/sltb.12703 33876483

[68] Eime RM, Young JA, Harvey JT, Charity MJ, Payne WR. A systematic review of the psychological and social benefits of participation in sport for children and adolescents: informing development of a conceptual model of health through sport. Int J Behav Nutr Phys Activity. 2013;10:1–21. 10.1186/1479-5868-10-98 PMC3751802 23945179

[69] Klonsky ED, Dixon-Luinenburg T, May AM. The critical distinction between suicidal ideation and suicide attempts. World Psychiatry. 2021;20(3):439. 34505359 10.1002/wps.20909 PMC8429339

[70] WHO. Social determinants of mental health. World Health Organization; 2014.

[71] Laursen B, Collins WA. Parent-child relationships during adolescence. 2009.

[72] Näher A-F, Rummel-Kluge C, Hegerl U. Associations of suicide rates with socioeconomic status and social isolation: findings from longitudinal register and census data. Front Psychiatry. 2020;10:898. 31992995 10.3389/fpsyt.2019.00898 PMC6971176

[73] Stanisławski K. The coping circumplex model: an integrative model of the structure of coping with stress. Front Psychol. 2019;10:694. 31040802 10.3389/fpsyg.2019.00694 PMC6476932

[74] Klimes-Dougan B, Mirza SA, Babkin E, Lanning C. Biased reporting of past self-injurious thoughts and behaviors: a literature review. J Affect Disord. 2022;308:596–606. 35429538 10.1016/j.jad.2022.04.073

[75] Allen J, Wexler L, Rasmus S. Protective factors as a unifying framework for strength-based intervention and culturally responsive American Indian and Alaska native suicide prevention. Prev Sci. 2021:1–14. 10.1007/s11121-021-01265-0 34169406

[76] Ati NA, Paraswati MD, Windarwati HD. What are the risk factors and protective factors of suicidal behavior in adolescents? A systematic review. J Child Adolesc Psychiatric Nurs. 2021;34(1):7–18. 10.1111/jcap.12295 33025698

[77] Keles B, McCrae N, Grealish A. A systematic review: the influence of social media on depression, anxiety and psychological distress in adolescents. Int J Adolescence Youth. 2020;25(1):79–93.

[78] Dat NT, Mitsui N, Asakura S, Takanobu K, Fujii Y, Toyoshima K et al. The effectiveness of self-esteem-related interventions in reducing suicidal behaviors: a systematic review and meta-analysis. Front Psychiatry. 2022:1219. 10.3389/fpsyt.2022.925423 PMC9240430 35782451

[79] Lindow JC, Hughes JL, South C, Minhajuddin A, Gutierrez L, Bannister E, et al. The youth aware of mental health intervention: impact on help seeking, mental health knowledge, and stigma in US adolescents. J Adolesc Health. 2020;67(1):101–7. 32115325 10.1016/j.jadohealth.2020.01.006 PMC7311230

[80] Kahn JP, Cohen RF, Tubiana A, Legrand K, Wasserman C, Carli V, et al. Influence of coping strategies on the efficacy of YAM (Youth Aware of Mental Health): a universal school-based suicide preventive program. Eur Child Adolesc Psychiatry. 2020;29(12):1671–81. 32025960 10.1007/s00787-020-01476-w

[81] Hale GE, Colquhoun L, Lancastle D, Lewis N, Tyson PJ. Physical activity interventions for the mental health and well-being of adolescents–a systematic review. Child Adolesc Mental Health. 2021;26(4):357–68. 10.1111/camh.12485 34105239

