# Supplementary material for: Protective factors for suicidal ideation and suicide attempts in adolescence: a longitudinal population-based cohort study examining sex differences
Source: BMC Psychiatry. 2025 Feb 6;25:106. doi: 10.1186/s12888-025-06552-6 (PMC11800530; doi:10.1186/s12888-025-06552-6)
Supplement: Supplementary file 1 — Supplementary Material 1 [file 12888_2025_6552_MOESM1_ESM.docx]

**Additional file**

**Table A1**
The distribution of suicidal ideation scale (0-2) by suicide attempt ordinal scores (0, 1, and 2) for females

| Suicidal ideation scale, mean scores (0-2) |  | Suicide attempt ordinal scores (0,1 and 2) | | |  | Total |
| --- | --- | --- | --- | --- | --- | --- |
|  |  | No | Yes, once | Yes, more than once |  |  |
| 0.00 |  | 797 | 11 | 0 |  | 808 |
| 0.20 |  | 152 | 11 | 3 |  | 166 |
| 0.25 |  | 2 | 0 | 0 |  | 2 |
| 0.40 |  | 61 | 6 | 2 |  | 69 |
| 0.60 |  | 30 | 5 | 3 |  | 38 |
| 0.75 |  | 1 | 0 | 0 |  | 1 |
| 0.80 |  | 26 | 9 | 2 |  | 37 |
| 1.00 |  | 11 | 4 | 5 |  | 20 |
| 1.20 |  | 10 | 1 | 0 |  | 11 |
| 1.40 |  | 8 | 4 | 1 |  | 13 |
| 1.60 |  | 7 | 4 | 1 |  | 12 |
| 1.80 |  | 1 | 6 | 2 |  | 9 |
| 2.00 |  | 1 | 4 | 6 |  | 11 |
| Total |  | 1107 | 65 | 25 |  | 1197 |

**Table A2**
The distribution of suicidal ideation scale (0-2) by suicide attempt ordinal scores (0, 1, and 2) for males

| Suicidal ideation scale, mean scores (0-2) |  | Suicide attempt ordinal scores (0,1 and 2) | | |  | Total |
| --- | --- | --- | --- | --- | --- | --- |
|  |  | No | Yes, once | Yes, more than once |  |  |
| 0.00 |  | 971 | 10 | 2 |  | 983 |
| 0.20 |  | 92 | 0 | 1 |  | 93 |
| 0.40 |  | 26 | 2 | 1 |  | 29 |
| 0.50 |  | 1 | 0 | 0 |  | 1 |
| 0.60 |  | 26 | 0 | 1 |  | 27 |
| 0.75 |  | 0 | 1 | 0 |  | 1 |
| 0.80 |  | 10 | 1 | 2 |  | 13 |
| 1.00 |  | 7 | 3 | 0 |  | 10 |
| 1.20 |  | 2 | 1 | 5 |  | 8 |
| 1.25 |  | 1 | 0 | 0 |  | 1 |
| 1.40 |  | 3 | 1 | 0 |  | 4 |
| 1.60 |  | 1 | 2 | 1 |  | 4 |
| 1.80 |  | 3 | 0 | 0 |  | 3 |
| 2.00 |  | 0 | 0 | 5 |  | 5 |
| Total |  | 1143 | 21 | 18 |  | 1182 |
